# Asiatic acid influences parasitaemia reduction and ameliorates malaria anaemia in *P. berghei* infected Sprague–Dawley male rats

**DOI:** 10.1186/s12906-016-1338-z

**Published:** 2016-09-13

**Authors:** G. A. Mavondo, B. N. Mkhwananzi, M. V. Mabandla, C. T. Musabayane

**Affiliations:** Discipline of Human Physiology, School of Laboratory Medicine, College of Health Sciences, University of KwaZulu Natal, Private Bag X54001, Westville Campus, Durban, 4000 South Africa

**Keywords:** Asiatic acid, Chloroquine, Malaria parasitaemia, *Plasmodium berghei*

## Abstract

**Background:**

Current malaria treatment is either “anti-parasitic”, “anti-infectivity” or both without addressing the pathophysiological derangement (anti-disease aspect) associated with the disease. Asiatic acid is a natural phytochemical with oxidant, antioxidant and anti-inflammatory properties whose effect on malarial and accompanying pathophysiology are yet to be investigated. Asiatic acid influence in *P. berghei*-infected Sprague Dawley rats on %parasitaemia and malarial anaemia were investigated.

**Methods:**

*Plasmodium berghei*-infected rats (90–120 g) were orally administered with Asiatic acid (5, 10, 20 mg/kg) and 30 mg/kg chloroquine as a positive control. Changes in %parasitaemia and haematological parameters in Asiatic acid administered rats were monitored in a 21 day study and compared to controls.

**Results:**

All animals developed stable parasitaemia (15-20 %) by day 7. Asiatic acid doses suppressed parasitaemia, normalised haematological measurements and influenced biophysical characteristics changes. Most positive changes were associated with intragastric administration of 10 mg/kg Asiatic acid dose. Peak %parasitaemia in Asiatic acid administration occurred at days 12 with a shorter time course compared to day 9 for chloroquine (30 mg/kg) treatment with a longer time course.

**Conclusions:**

Oral Asiatic acid administration influenced %parasitaemia suppression, ameliorated malarial anaemia and increased biophysical properties on infected animals. Asiatic acid may be a replacement alternative for chloroquine treatment with concomitant amelioration of malaria pathophysiology. Due to different action time courses, Asiatic acid and chloroquine may be possible candidates in combination therapy.

## Background

One or more of the five *Plasmodium* species known to infect human beings cause malaria accounting the death of over 600, 000 people annually, a majority of which are pregnant women and children less than 5 years of age [1]. The pathophysiology of malaria, which is the “malaria disease”, include immunological aberrations, inflammation, haemolysis with severe malaria anaemia [SMA] [2], acute renal failure and general cachexia [3] and malaria cachexia leading to cardiac failure [4]. Pathophysiological manifestations during or after successful treatment of infection are major causes of high morbidity and mortality associated with malaria [5]. Untoward post treatment effects with artemisinins and chloroquine and drug resistance affect the mono therapeutic usage of these historical drugs [6]. Novel anti-inflammatory and immunoregulatory functions of artemisinin and its derivatives has been reported to inhibit nitric oxide (NO) and proinflammatory cytokines production by suppressing MRPK and NF-kβ in macrophages cell line RAW 264.7 [7]. However, the continued need of for antipyretic supportive paracetamol (acetaminophen) therapy prolongs parasite clearance time by decreasing induced tumour necrosis factor-α (TNF-α) and may worsen the disease with its inadequate “anti-disease effects” necessitating continuing search for more effective “anti-parasitic” or “anti-infection” or both.

Efforts to use adjunctive therapy in malaria, born out of the identification of malaria pathophysiology resolution was key to treatment of the disease, have not managed to reduce malaria morbidity and mortality. Dexamethasone (steroid), antibodies against TNF-α, phenobarbital (anti-convulsant) and iron chelation with desferrioxamine administration in malaria have not yielded expected outcomes. Therefore, there still exists an acute need for antimalarial drugs with anti-parasitic, anti-infectivity and anti-disease properties. Aberrant immune response and uncontrolled inflammatory process driven by increasing parasitaemia in malaria, formulate a significant part of a vicious cycle in a feed-forward mechanism leading to severe malaria anaemia, general cachexia, coma and death from the disease [8]. Hypothetically, drugs that may inhibit or reverse malaria pathophysiology or the disease components have a higher chance of controlling malaria even without parasite eradication through targeting growth essential host related factors such as severe malaria anaemia [SMA] an independent malaria mortality predictor in pregnant women and children. We hypothesized that triterpenes with anti-disease properties in other conditions similar to malaria, like inflammation in sepsis and hypoxia in anaemia may be able to eradicate the *Plasmodium* parasite as well as resolve the ensuing pathophysiology.

Triterpenes with pleiotropic functions, sufficient to be anti-disease as well as anti-parasitic have been reported. Betulinic acid [BA] (IC_50_ 19.6 and 25.9 μg/mL), ursolic acid [UA] (IC_50_ 36.5 and 28 μg/mL) and oleanolic acid [OA] (88.8 and 70.6 μg/mL) have been shown to have moderate activity in vitro against the chloroquine insensitive (K1) and chloroquine sensitive (T9–96) *Plasmodium falciparum* parasites [9]. Maslinic acid, a possible multi targeting antimalarial, effectively inhibited proteolytic processing of the merozoite surface protein (MSP1) complex, inhibited the metalloproteases and revealed (in silico studies) two targets while suggesting several putative new binding sites for the natural triterpene [10]. This multi-target phenomenon suppresses the parasitaemia and avoids the age old preoccupation with targeting single process of the parasite infective cycle (which is mutation prone) to involve host-related responses potentiating anti-disease and anti-resistance outcomes.

Asiatic acid (AA), an amphiphilic triterpene, with known antioxidant and pro-oxidant capacity [11], anti-inflammatory and antinociception activity in mice [12], calcium-release associated apoptosis induction [13] and a potent immunomudulator shares structural and bioactivity properties with OA, MA, UA and BA making it a possible antimalarial agent. Indeed, prophylaxis activity of AA has recently been suggested [14] together with its influences on glucose homeostasis in malaria [15].

Proper interventions may inhibit malarial pathology development which should be the aim of malaria management seeing that people living in endemic areas develop partial to total immunity against the parasite and asymptomatic parasitaemia is common [16]. Therefore, targeting the pathophysiology of malaria as well as the parasite may provide a new mechanism of combating malaria. It is noteworthy that the diseases and conditions AA is known to attenuate, inhibit or ameliorate formulate the bedrock of malaria disease and sequalae as alluded earlier [2, 3, 17–19]. However, there is little information on the antimalarial, haematological, immunological impact of AA in malaria, facets that require exploration for possible AA pharmacotherapeutic uses. Malaria is driven by glycosylphosphatidylinositol (GPI) which elicits excessive macrophages activation leading to proinflammatory cytokine release, tissue damage, erythophagocytosis, erythropoiesis dysfunction and general cachexia, require an animal model to unravel the complex disease pathophysiology [20]. AA modulates immunity by selective induction of mitochondria-dependent apoptosis of activated lymphocytes in the prevention of murine fulminant hepatitis [21] a mechanism that may be extendable to malaria. Using membrane DNA array technique, a wound-healing derivative of AA [2-Oxo-3, 23-isopropylidene-asiatate (AS2006A)] exerted anti-inflammatory effect through selective cytotoxicity to activated macrophage cell line (L-929) by upregulating expression of apoptosis-inducing genes caspase-8, c-myc, inducible nitric oxide synthase (iNOS), mdm2, NF-kβα, I-kβα, and NF-kβ p105 [22]. The finding may allude to AA also exerting anti-inflammatory effect by cytochrome c release, caspase 3 activation and poly(ADP-ribose) polymerase cleavage mechanism as did AS2006A which may require possibly elucidation in vivo experiments.

The effect of AA in alleviating haemodynamic and metabolic alterations in rats with metabolic syndrome through restoration of endothelium nitric oxide synthase (eNOS)/iNOS expression [23] has been reported. Similar AA influence in murine malaria may be anticipated where eNOS/iNOS ratio determines the bioavailability of NO necessary for proliferation and angiogenesis [24]. Derangements of this relationship may formulate malarial microvascular pathology.

Limitations inherent in in vitro studies and the inaccessibility of the human host further navigates research of this nature to an animal model with works by Thaane, T on maslinic acid (MA) [25] and by Mbatha B OA [26] in the murine malaria animal model suggesting amelioration of pathophysiological derangements in malaria. Furthermore, *Centella asiatica*, from which naturally occurring AA is obtained, was used to treat malaria related fevers [27] although no specific mention of the phytochemical has been associated with malarial treatment. In vitro studies, whilst they may show the molecular interaction of AA with the parasite, they fall short where the pathophysiology of the disease are being explored as in this current study. In recognition of this, AA antimalarial and its malaria pathophysiology ameliorative effects were investigated in *P. berghei*- infected young (90–120 g) male Sprague Dawley rats as a novel effect of AA in malaria.

## Methods

### Materials

#### Drugs, chemical and accessories

AA (97 % purity), chloroquine diphosphate and dimethyl sulphoxide (DMSO) were purchased from Sigma-Aldrich (St. Louis, Missouri, USA). All other chemicals and reagents used were of analytical grade.

#### Animals

Male Sprague–Dawley (SD) rats (90–120 g) were obtained from the Biomedical Research Unit (BRU), of the University of KwaZulu-Natal where they were bred and housed for the entire experiment period. Animals were housed communally and individually in Makrolon polycarbonate metabolic cages (Techniplast, Labotec, South Africa) during experiments. The animals were kept under maintained laboratory conditions of constant temperature (22 ± 1 °C); C0_2_ (<5000 ppm), humidity of 55 ± 5 % and illumination (12 h light/dark cycles) with access to standard rat chow (Meadows Feeds, Pietermaritzburg, South Africa) and water ad libitum. All animals were sacrificed by day 21 through exposure to lethal anaesthetic inhalation of isofor (Safeline Pharmaceuticals, Rooderport, South Africa) for 3 min via an anaesthetic gas chamber (100 mg/kg). All experiments and protocols used in this study were reviewed and approved by the animal ethics committee of the University of KwaZulu Natal (UKZN) with ethical clearance numbers 079/14/Animal and 013/15/Animal issued.

#### Plasmodium parasite

Chloroquine-susceptible strain of *Plasmodium berghei* ANKA, murine malaria parasite was a kind donation from Professor Peter Smith (University of Cape Town, Division of Clinical Pharmacology, South Africa). The parasite was sub-cultured, harvested and stored in a Bio Ultra freezer (Snijers Scientific, Tilburg, Netherlands) at −80 °C until use.

#### Experimental design

The study was conducted over 21 day in animal groups (*n* = 6 per group) as follows:

- Non-infected treated control groups (NIC)
- Infected non-treated control groups (IC)
- Infected treated with CHQ 30 mg/kg groups (30CHQ)
- Infected administered AA 5 mg/kg groups (5 mg)
- Infected administered AA 10 mg/kg groups (10 mg)
- Infected administered AA 20 mg/kg groups (20 mg)

### Methods

#### Induction of parasitaemia

Chloroquine-susceptible strain of *P. berghei* ANKA (10^6^ parasitized red blood cells [pRBC’s] suspension in saline) was inoculated intraperitoneal (ip). Control animals received equivalent amount of saline. Day of inoculation was regarded as experiment day 1.

#### Oral Asiatic acid and chloroquine preparation

Asiatic acid was dissolved in DMSO (0.5 mL) and made up to volume with distilled water such that the final concentration was 5 mg/kg AA. Dosage was administered as multiples of the stock volume equivalent to the dose required. Chloroquine [CHQ] (30 mg/kg) was dissolved in distilled water. Both compounds were prepared fresh each day.

#### Monitoring of %parasitaemia

Appearance of parasites in the blood after ip inoculation takes 2–3 days [28]. Pre-patent period of 72 h post infection and a stable state parasitaemia at 15–20 % on day 7 without intervention confirmed the models conform to the known murine malaria course. These periods can be used as set points for establishing predictive validity of the experiments. %Parasitaemia was assessed and measured on day 3 and 7 in infected groups. Treatment was only commenced when the %parasitaemia had reached patent level or stable state SM.

#### Influence of Asiatic acid on biophysical changes

In animals individually housed in metabolic cages, intake of food, water and body weights of non-treated infected (IC), non- infected treated (NIC) and infected treated were monitored gravimetrically at 09 h00 every third day for experiment duration. The effects of AA on the measured parameters were compared to those of controls.

#### Malaria treatment

Rats were treated per oral (po) using a ball-tipped, 18-gauge gavage needle (Kyron Laboratories (Pty) LTD, Benrose, South Africa) attached to a 1 ml syringe. Due to preliminary studies on AA posology and literature reports AA (5, 10, 20 mg/kg) [29] was administered on day 7–12 (5 days), once daily at 09 h00. CHQ (30 mg/kg) was administered twice daily (09 h00 and 16 h00) according to local laboratory developed posology [25, 26]. The CHQ dose of 30 mg/kg was selected although it was larger than the highest dose of AA (20 mg/kg) because it is treatment dose lethal to *P. berghei* which is closest to AA doses in the study that has been used by other researchers [30].

#### Influence of AA administration on % parasitaemia

Peripheral (tail) blood smears were used to monitor %parasitaemia. The actual number of pRBC’s relative to 2 × 10^4^ RBC’s was used to calculate parasitaemia [28]. A 15–20 % parasitaemia, confirmed by Giemsa staining under a microscope (Olympus Cooperation, Tokyo, Japan), was considered as stable state of severe malaria (SM). For post-infection AA or CHQ po administration % parasitaemia was monitored at 72 h (pre-patent period), every third day up to Day 7 [patent period] [31], every day during treatment period (5 days) and thereafter every other day post-treatment period until day 21.

#### Influence of AA administration on malaria anaemia

Changes in full blood count (FBC) were used to further confirm the influence of AA in reversing low blood volume experienced in malaria. Animals were sacrificed on days 0, 8, 12, 21 and blood was collected by cardiac puncture into EDTA tubes for full blood count analysis.

### Statistical analysis

Unless otherwise stated, data was presented as mean ± standard error of the mean (SEM). Statistical comparisons were performed by one way analysis of variance (ANOVA), followed by Tukey-Kramer multiple comparison post hoc test using GraphPad InStat Software (version 5, GraphPad Software, San Diego, California USA). A *p* < 0.05 considered statistically significant.

## Results

### Asiatic acid influence in malaria

#### Per-oral AA administration on %parasitaemia

**Fig. 1:**
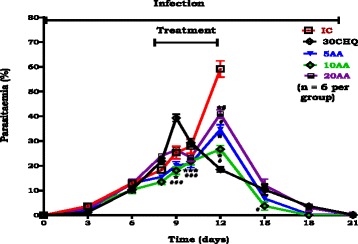
Influence of oral AA on % parasitaemia compared to controls. IC is infected-non treated control. 30CHQ is chloroquine 30 mg/kg. Values are presented as means ± SEM, (*n* = 6 in each group). *, # *p* < 0.05 compared to IC, CHQ controls, respectively

Figure 1 shows %parasitaemia changes over time. AA (5, 10, 20 mg/kg) decreased %parasitaemia in comparison to IC (*p < 0.05) during treatment. AA (5, 10, 20 mg/kg) administration varied significantly vs 30CHQ on days 8–12 (#*p* < 0.05). AA (10 mg/kg) decreased %parasitaemia significantly in comparison to 30CHQ (#*p* < 0.05) post treatment period day 15. Peak parasitaemia had significantly different time points when AA (5, 10, 20 mg/kg) administered groups were compared to 30CHQ (#*p* < 0.05) over the 21 day study.

#### AA effects on %parasitaemia-time area under the curves

**Fig. 2:**
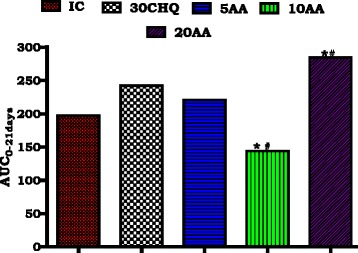
Influence of AA administration on haemoglobin changes over time. NIC is non infected treated control; IC is infected non treated control; 30CHQ is chloroquine treated with 30 mg/kg. Values are presented as means ± SEM, (*n* = 6 in each group). α,*, # *p* < 0.05 compared to NIC, IC, CHQ controls

%parasitaemia-time course, indicated by area under the curve (AUC), compared the cumulative effect of different AA doses on the parasite and malaria pathophysiology to those of IC (AUC_0-12days_) and CHQ (AUC_0-21days_) as shown in Fig. 2. Suppression efficiency and malaria pathology amelioration of different doses of AA (AA5, 10, 20 mg/kg UC_0-21days_) was in the order of 10 mg/kg > 5 mg/kg > 20 mg/kg. AA 10 mg/kg administration lowered the AUC_0-21days_ significantly compared to the IC AUC_0-12days_ and 30CHQ treatment AUC_0-21days_ (*, # *p* < 0.05, respectively). AA 20 mg/kg administration had an increased AUC_0-21days_ compared to both the IC AUC_0-12days_ and 30CHQ AUC_0-21days_ (*, # *p* < 0.05, respectively). There was no significant difference in %parasitaemia-time course of AA5 (AUC_0-21days_) when compared to CHQ (AUC_0-21days_) and IC (AUC_0-21days_).

#### Influence of AA on parasitaemia progression compared to controls

Table 1 showed %parasitaemia-time lines which revealed that AA doses influence varied according to the different doses when compared to controls. At day 7 AA20 had significantly lower %parasitaemia compared to CHQ (*p < 0.05) while other doses did not show significant differences to controls. At day 7 AA10 displayed a higher %parasitaemia compared to IC and CHQ (*, #*p* < 0.05, respectively). At peak % parasitaemia AA10 administration had a higher parasitaemia suppression effect compared to controls (*, #*p* <0.05.) AA5 and 20 had equally significant %parasitaemia suppression capacity at peak parasitaemia compared to CHQ (#*p* < 0.05). %parasitaemia progression was faster in AA10 administered animals. From day 3 to day 7 while it was slowest in AA20 administered animals.

**Table 1:** Asiatic acid influence on parasitaemia % time lines compared to controls

Protocol	Dose	Infection rate	Pre-patent (days)	% parasitaemia day 3	% parasitaemia range on Day 7	% peak parasitaemia
Post-Infection per oral treatment	IC	100 %	2–3	3.70 ± 0.675	15.7 ± 3.264	69.3 ± 3.88
	30CHQ	100 %	2–3	1.167 ± 0.307	16.1 ± 1.325	43.3 ± 1.66
	5AA	100 %	2–3	1.928 ± 0.778	16.9 ± 2.774	41.7 ± 4.51*
	10AA	100 %	2–3	3.230 ± 2.209	21.3 ± 3.608*#	27.7 ± 1.96*#
	20AA	100 %	2–3	1.815 ± 1.016	8.4 ± 3.848*#	46.2 ± 2.94*

*IC* infected-non treated control, 30CHQ is chloroquine 30 mg/kg. Values are presented as means ± SEM. (*n* = 6 in each group)

### Influence of Asiatic acid on eating and drinking habits as well as weight changes

#### Influence of AA on biophysical properties

Food and water intake plus weight changes reflected the general animal health among AA treated infected animals compared to controls. Tables 2, 3 and 4 showed the effects of AA on food and water intake, and %weight change. At day 12 and 21 food intake of AA administered animals increased in order of 5 mg/kg > 20 mg/kg > 10 mg/kg vs IC and 30CHQ (*p < 0.05 and #*p* < 0.05, respectively). Administration of 5 and 20 mg/kg AA significantly (*p < 0.05) increased food intake by comparison to the IC on day 7–12 of treatment. AA administration (5, 10, 20 mg/kg) significantly increased water intake when compared to IC and 30CHQ group (*p < 0.05 and #*p* < 0.05, respectively) at patent/treatment and post treatment time points. Animals administered AA (5, 10, 20 mg/kg) had increased %weight change in comparison to IC and 30CHQ (*, #*p* < 0.05, respectively) during treatment and post treatment periods. Overall the 10 mg/kg AA had the highest positive effect on biophysical changes by comparison to all controls (*, #*p* < 0.05). Generally, AA administration had a positive influence on the biophysical characteristics of the experimental animals compared to controls.

**Table 2:** Post-infection AA5, 10, 20 oral administration influence on food intake changes compared to controls

Parameter	Post-infection treatment	Pre-patent (D 3)	Patent/Treatment (D7–12)	Post-treatment (D13- 21)
Food intake (g/100 g)	NIC	12.3 ± 2.3	13 ± 9.0	13.4 ± 1.6
	IC	11.5 ± 1.9	6.7 ± 1.3	N/A
	30CHQ	12.3 ± 1.8	7.8 ± 2.0	8.6 ± 1.7
	5 AA	12.9 ± 1.3	9.8 ± 1.3*#	9.7 ± 1.5*#
	10 AA	12.3 ± 1.8	12.9 ± 2*#	13.2 ± 0.72#
	20 AA	12.7 ± 1.6	9.2 ± 1.6α*	10.3 ± 1.2#

NIC is non-infected treated control, IC is infected non-treated control and CHQ is chloroquine control (30 mg/kg). Values are presented as means ± SEM, (*n* = 6 in each group). α,*,#*p* < 0.05 by comparison with NIC, IC, 30CHQ groups, respectively. *NIC* non infected treated control, *IC* infected control, *CHQ* chloroquine, *AA* asiatic acid

**Table 3:** Post-infection AA5, 10, 20 oral administration influence on water intake changes compared to controls

Parameter	Post-infection treatment	Pre-patent (D 3)	Patent/Treatment (D7–12)	Post-treatment (D13- 21)
Water intake (mL/100 g)	NIC	14.3 ± 1.0	15.5 ± 1.3	15.2 ± 0.9
	IC	15.5 ± 1.3	8.4 ± 1.7	N/A
	CHQ	14.2 ± 1.2	10.5 ± 1.7	12 ± 1.2
	5AA	15 ± 3	12.7 ± 1*#	14.8 ± 1.9#
	10AA	15.8 ± 0.8	14.2 ± 0.9*#	15.1 ± 1.1#
	20AA	15.2 ± 1	11.6 ± 1.7α*	12.9 ± 1.6

NIC is non-infected treated control, IC is infected non-treated control and CHQ is chloroquine control (30 mg/kg). Values are presented as means ± SEM, (*n* = 6 in each group). α,*,#*p* < 0.05 by comparison with NIC, IC, 30CHQ groups, respectively. *NIC* non infected treated control, *IC* infected control, *CHQ* chloroquine, *AA* asiatic acid

**Table 4:** Post-infection AA5, 10, 20 oral administration influence on % weight change changes compared to controls

Parameter	Post-infection treatment	Pre-patent (D 3)	Patent/Treatment (D7–12)	Post-treatment (D13- 21)
% body weight change	NIC	8 ± 1	14.4 ± 6	18.9 ± 6
	IC	10.1 ± 5	4 ± 22.3	N/A
	CHQ	8.8 ± 1.5	9.4 ± 6	12.2 ± 0.5
	5AA	9.3 ± 2.2	14.2 ± 1.0*#	15.5 ± 1.9#
	10AA	8 ± 1	13.7 ± 1*	17.3 ± 0.8#
	20AA	9.5 ± 1	11.5 ± 1.9*	13.1 ± 0.2

NIC is non-infected treated control, IC is infected non-treated control and CHQ is chloroquine control (30 mg/kg). Values are presented as means ± SEM, (*n* = 6 in each group). *, #*p* < 0.05 by comparison with IC and 30CHQ groups, respectively. *NIC* non infected treated control, *IC* infected control, *CHQ* chloroquine, *AA* asiatic acid

### Influence of AA on anaemia development and resolution

#### Influence of AA administration on haemoglobin

**Fig. 3:**
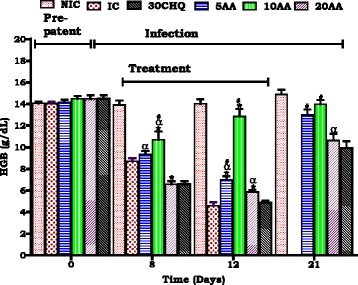
Influence of AA administration on haematocrit changes over time. NIC is non infected treated control; IC is infected non treated control; 30CHQ is chloroquine treated with 30 mg/kg. Values are presented as means ± SEM, (*n* = 6 in each group). α,*, #*p* < 0.05 compared to NIC, IC, CHQ controls

Hb concentration estimated the degree of anaemia development and resolution compared to controls. Figure 3 AA (10 mg/kg) vs NIC, day 7–12 (α *p* < 0.05). AA (10 mg/kg) vs IC on days 7–12 (*p < 0.05). AA (10 mg/kg) vs 30CHQ on day 7–12 and post treatment (# p0.05). AA (5 mg/kg) vs NIC on days 7–12 (α *p* < 0.05). AA (5 mg/kg) vs IC on days 7–12 (*p < 0.05). AA (20 mg/kg) vs NIC on days 7–12 and post treatment (α *p* < 0.05). AA (20 mg/kg) vs IC on days 7–12 (*p < 0.05). AA (20 mg/kg) vs 30CHQ on days 7–12 and post treatment (# *p* < 0.05).

#### Influence of AA administration on haematocrit compared to controls

Haematocrit change over time was used as a surrogate marker for severe malaria anaemia development and resolution. Compared to the IC, AA10 administration was shown to influence a higher haematocrit at patent/ treatment periods (*p < 0.05). AA5, 10, 20 administration had generally lower haematocrit during patent/treatment and post treatment periods compared to NIC (α *p* < 0.05). AA10 administration influenced higher haematocrit compared to CHQ (# *p* < 0.05). AA10 administration proved to be the most efficacious of the three doses against malaria anaemia development.

#### Influence of AA administration on red blood cell mass compared of controls

Red blood cell mass was used as a critical indicator of anaemia development and resolution Compared to the IC, AA10 administration was shown to influence a higher red cell count at patent/ treatment periods (*p < 0.05). AA10 administration influenced higher red cell count compared to CHQ (# *p* < 0.05). Compared to NIC patent/treatment and post treatment period AA administration had lower red cell count. AA10 administration proved to be the most potent of the three doses in protecting red cell count.

## Discussion

There is paucity of information with regard to AA treatment of malaria although anti-malaria anecdotal evidence has been forwarded for *Centella asiatica* [CA] from which AA is obtained. *P. berghei* has a similar pathophysiology with the *P. falciparum,* the most virulent human malaria parasite warranting its use as a murine malaria model. Studies in our laboratory have indicated, also proven in earlier research [32], that *P. berghei* causes severe malaria (SM) in younger animals which may proceed to cerebral malaria (CM).

The *P. berghei*-SD rat malaria model conformed to a high predictive validity at days 3 and 7 (Table 2) as expected. Infection effectuation by an ip inoculation of 10^6^ parasitized red blood cells (pRBC’s) saline suspension invariably resulted in SM. There was no significant difference in %parasitaemia observed amongst the groups during the pre-patent and patent periods. Decrease in %parasitaemia and changes in haematological parameters, therefore, could be attributable to the intervention.

**Fig. 4:**
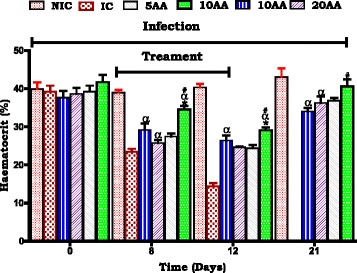
Influence of AA administration on red blood cell count compared to controls. NIC is non infected treated control; IC is infected non treated control; 30CHQ is chloroquine treated with 30 mg/kg. Values are presented as means ± SEM, (*n* = 6 in each group). α,*, #*p* < 0.05 compared to NIC, IC, CHQ controls

We used young, 6 weeks old SD rats (90–120 g) which displayed SM, severe malaria anaemia (SMA) and in some cerebral malaria (CM) to demonstrate AA influence on murine malaria, SMA development and its resolution. Stable state SM was determined as 15–20 % parasitaemia at day 7 and >20 % at day 12 (Fig. 1) and SMA as an Hb <7.0 g/dL, Hct <15 % and red cell count <4 × 10^6^/μL (Figs. 2, 3, 4).

In untreated animals peak parasitaemia was able to reach 69.3 % (Table 2) and Hb was 4.5 g/dL (Fig. 2) by day 12 when the animals were sacrificed to avert further pain and suffering. Suppression of parasitaemia to undetectable levels and resolution of haematological parameters to normal by administration of AA10mg/kg by day 21 was demonstrated. Once daily administration of AA10 corrected malaria manifestations compared to the IC and 30CHQ (twice daily treatment).

**Fig. 5:**
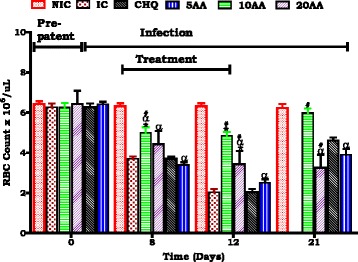
AUC_0-21Days_ of %parasitaemia for post-infection AA oral administration. IC is infected-non treated control. 30CHQ is chloroquine 30 mg/kg. Values are presented as means ± SEM, (*n* = 6 in each group). *, # *p* < 0.05 compared to IC, CHQ controls, respectively

Partially pharmacodynamics of AA in influencing malaria was shown by comparing the efficacy of the different AA doses during the 21 day studies. Normally, a drug at higher concentrations is expected to exert a higher and more profound an effect and to be less effective at lower concentrations. While we did not determine the minimum inhibitory concentration for AA in this study, at highest (AA20) and lowest (AA5) doses, AA po administration may have been permissive to parasite growth through currently unknown mechanisms by displaying higher peak %parasitaemia comparable to CHQ although significantly lower than the IC (Table 2). AA5, AA20 and 30CHQ permitted %parasitaemia to increase by 1.47, 4.5 and 1.69 times, respectively, from day 7 to peak %parasitaemia at day 21 compared to AA10 which increased by 0.3. It may be poignant to note that AA20 had the highest effect on parasitaemia suppression during early days of AA administration, thereafter %parasitaemia dramatically increased subsequently culminating in the highest %parasitaemia- time course (Figs. 1, 5 and Table 2). Administration of AA5 may not have reached an optimum drug concentration to be lethal to the parasite during the treatment period while the AA20 dose may have been too high becoming protective to the parasite’s survival over time. Administration of AA10 provided the optimum dose as the suppression of %parasitaemia was evidenced by a higher patent/treatment period %parasitaemia without dramatic increase towards peak %parasitaemia.

The %parasitaemia-time under the curve (AUC) displayed the cumulative effect of the different AA doses collated over 21 days for AA, CHQ and over 12 days for the IC. Although the %parasitaemia-time course for IC (IC AUC_0-12days_) was shorten to 12 days, compared to 21 days for the treated animals, still the latter had a comparatively higher AUC showing that AA intervention resulted in lowering of AA10 AUC_0-21days_ as observed. Indeed, AA10 administration resulted in a lower AUC_0-21days_ showing a more potent parasitic suppression effect of AA at this dose.

The observed continual %parasitaemia decline beyond AA administration at day 12 implies possible residual activity of AA with three possible effects: an accumulative AA concentration with increased direct parasite metabolism disruption or increased malarial pathophysiology resolution hostile to parasite survival or immune system activation. A combination of these and other phenomena is possible as well. However, this dynamic milieu, which may be explainable through recognition of the molecular characteristics of AA, was only attained by AA10 administration.

The phytochemical AA possesses both antioxidant (hydrogen bond acceptor 4.172) and pro-oxidant (hydrogen bond donor 7.1) capacities [11] which may concurrently function under physiological conditions and may eventually increase during subsequent dosing with possible chemoprophylaxis and chemotherapeutic effects. Hypothetically, the ratio of the oxidant and antioxidant capacity may dictate which facet of AA predominates. To the parasite, an oxidative interaction with AA will be detrimental to its survival while an antioxidant may be beneficial. There is a possibility that the optimum redox equilibrium of AA with a parasite killing potential was obtainable only at AA10 dose while it was diminished at AA20 and AA5 doses with resultant parasite proliferation seen by day 12.

In malaria, inflammatory response is usually exaggerated. The host’s innate immune response to contain parasitaemia invariably involves the production and release of Th1 and Th2 cytokines, chemokines, growth factors, inflammatory effectors and mediators which throw the regulatory process off balance resulting in anaemia and other malaria pathology. AA has anti-inflammatory activity which could have resolved this pathophysiology, weakening malaria virulence and suppressing parasitaemia when AA10 was administered. Furthermore, the immunomodulatory effect of AA10 may also have curtailed aberrant immune reactivity that is common in malaria by selectively suppressing activated lymphocytes [21] and macrophages proliferation with possible enhancement erythroid lineage propagation. An intricate balance in the host’s eradication of the parasite exists during malaria proliferation through the production of inducible tumour necrosis factor (TNF), oxygen free radicals and other inflammatory mediators [33]. This could have implied that the anti-inflammatory and immunomodulatory effects of AA20 could have prolonged parasitaemia through over enhancement of these characteristics.

These the parasite suppression effects or lack off may also have been facilitated by a dose dependent AA absorptivity in the small intestines such that higher plasma concentrations were achieved through higher AA dose administration. Indeed, AA has been predicated to have a good intestinal absorption, mild Caco-2 cell permeability and a strong plasma protein binding [34] and low excretion. The strong protein binding of AA may have caused possible cumulative concentrations of the compound in plasma reaching a certain threshold that overwhelmed parasite defences with rapid parasitaemia decline. Moreover, a high affinity for proteins and low excretion rate may mean sustained, slow and constant release allowing AA to reach targets at optimum concentrations influenced by protein plasma concentrations which may be dependent on food and water intake.

Together with increased suppression of parasitaemia, administration of AA10 dose was associated with improved food and water intake and preserved weight gain compared to both 30CHQ dose and IC (Table 1). By preserving food and water intake at previous levels prior to infection, AA10 administration rebuffed onset of the sickness behaviour associated with malaria infection seeing that a significantly higher %parasitaemia was reported at day 7 (Table 2) showing infection patency. Malaria treatment with current drugs like CHQ does not counter parasite induced satiate but might actually exacerbate it. The decreased food and water intake and subsequently low weight gain in animals treated with CHQ could have been due a combined effect of both the parasite and the drug.

Chloroquine, like quinine, is known to have bitter taste which has been shown to reduce appetite [35]. The intestinal lining has receptors for both sweet and bitter substances with the latter associated with influencing decreased food intake through the gut-brain axis [36]. Nausea and vomiting are some side effects of CHQ which may increase aversion to food and water intake with worsening malarial pathophysiology, depletion of energy stores and decreasing foraging capacity. In this study, AA10 administration maintained food and water intake possibly due to its being tasteless and its antimalarial activity.

Furthermore, the hypoglycaemic effects of AA have been reported in streptozotocin-induced diabetes mellitus where the phytochemical increased activities of glycolytic enzymes while inhibiting gluconeogenesis and glycogenolysis [37]. Such an influence occurring in a normoglyaemic situation may invariably upregulate the energy mobilization processes to keep pace with the increased glucose utilization and demand of malaria. With food and water available ad libitum, an increased appetite as induced by AA10, physiologically inclines the animals to increase food and water intake. Therefore, it is plausible to assume that in malaria, a disease that influences induction of hypoglycaemia, AA10 had a causal relationship with weight gain and malaria complications alleviation.

One of the malaria sequalae that was observed to be averted is severe malaria anaemia (SMA). In malaria, SMA has multifaceted aetiologies ranging from parasitized red blood cells (pRBC’s) rapture, pRBC’s and non-parasitized red blood cells (npRBC’s) phagocytosis [31], insufficient erythropoiesis, ineffective haematopoiesis and reduced erythropoietin production. While the haematological indices of haemoglobin (Hb) concentration, red blood cell count (RBC’s) and haematocrit (Hct) were depressed with increasing %parasitaemia in the IC and CHQ controls, AA10 administration preserved these parameters and ameliorated SMA. SMA has been shown to persist even when parasitaemia has been resolved driven by an aberrant immune system and hemozoin-induced oxidative stress [38]. Therefore, current finding of AA10 parasitaemia suppression may not have been the only effect retarding and correcting SMA. There is possibility that more factors (including inflammatory mediators- impaired erythropoiesis) influencing SMA development may have been inhibited by AA10 per se together with preserved food and water intake.

Destruction of pRBC’s occurs when the shyzoints mature and merozoites rapture cell membranes. Accompanying pRBC’s destruction is the lysis of npRBC’s at a ratio of 8.5 RBC’s for each pRBC’s haemolysed [39]. Anaemia develops as the RBC mass is reduced rapidly without concurrent replacement. Endogenous secretion of erythropoietin (EPO) may be overwhelmed by the high demand for erythropoiesis stimulation that is required to meet the rapid development of anaemia during the patent period. However, it was also observed that increasing EPO was not able to alleviate SMA associated with high parasitaemia showing that erythroid precursor response may also be inhibited by blood stage parasites resulting in low reticulocytosis [40]. Erythroid progenitor suppression in malaria has been ascribed to the increased free haemoglobin and hemozoin containing monocytes in the bone marrow and a shift of the transferrin receptor expression from erythroid to non-erythroid cell to increase immunological response to blood-stage parasite [41].

Alleviation of SMA involves the generation of reticulocytes, a process which requires the proliferation, differentiation and maturation of erythrocyte precursors in the bone marrow and other haematopoietic tissues. The maintenance of RBC’s count in this study may have implied that there was sufficient reticulocytosis or there was minimum RBC’s destruction in AA10 administered animals compared to controls. However, the decrease in Hb and Hct at patent in IC and CHQ groups shows RBC’s destruction as one of the key initiators of SMA which was minimized by AA10 administration.

The mechanism by which npRBC’s are destroyed in malaria involves increased erythrocytic oxidative stress and parasite antigens which cause RBC’s membrane to be less deformable and more fragile with shortened RBC’s life spans. These cells are trapped during splenic sequestration and destroyed through phagocytosis. AA has known antioxidant, anti-inflammatory and immunomodulatory properties which could have protected cell membranes from oxidative damage and rigidity, reduced erythrophagocytosis and inhibited parasite proliferation. Masilinic acid (MA) a phytochemical similar in structure and polypharmacology with AA was shown to have multi-targeted inhibitory properties against malaria with possible blockade of parasite maturation from early ring to schizont stages [42]. Betulinic and ursolic acids also share carbon skeleton with MA and AA. Analogues of these two triterpenes have been shown to be antiplasmodial through disruption of parasite calcium homeostasis [43]. There is a possibility that AA may also possess the same inhibitory properties which could have limited RBC’s haemolysis and preserved haematological indices.

Fully functional murine haemoglobinised RBC’s take 7 days to be formed from an erythroid progenitor. Haematological indices in AA10 administered animals reflected the same time span for their recovery to near pre-infection levels showing an effectuation of a multi-factorial remedy to SMA by AA10. The pleiotropic biological effect of AA may have influenced host control of the parasite through modulation of the immune system erythropoiesis suppressive effect resulting in the haematological indices correction towards normal in AA10 administered animals compared to controls. The involvement of cytokines and inflammation mediators, such as haeme-parasite derived hemozoin, influence the differentiation and maturation of erythroid cells. Increased levels of tumour necrosis factor (TNF), interleukin 6 (IL6), interleukin 1β (IL1β) and decreased levels of interleukin 12 have been associated with SMA [44]. The persistence of SMA beyond parasitaemia eradication is orchestrated and sustained by an immunological sequalae which upregulates hepcidin synthesis and modulation of iron metabolism [45]. Therefore, resolution of SMA (normalised haematological indices) indirectly indicated the abrogation of the immunological and inflammatory processes by AA10, which properties the triterpene is known to possess. However, the exact mechanism by which AA10 modulated SMA development and resolution requires further exploration.

## Conclusion

Data demonstrating that AA positively influences food and water intake, %weight gain, % parasitaemia and SMA in *P. berghei*-infected SD rats has been presented. AA may have both anti-parasitic and anti-disease activities suppressing the parasite while ameliorating infection-induced pathology. AA10 administration showed a superior efficacy in preservation of food and water intake, parasitaemia suppression as well as resolution of SMA.

## Abbreviations

AA: Asiatic acid
CA: *Centella* asiatica
CHQ: Chloroquine
DMSO: Dimethyl sulphoxide
GPI: Glycosylphosphatidylinositol
IC: Infected non treated control
ig: Intragastric
Ip: Intraperitoneal
NIC: Non infected treated control
po: Per oral
SMA: Severe malaria anaemia
